# White blood cells and chronic rhinosinusitis: a Mendelian randomization study

**DOI:** 10.1186/s13223-022-00739-2

**Published:** 2022-11-22

**Authors:** Thanai Pongdee, Suzette J. Bielinski, Paul A. Decker, Hirohito Kita, Nicholas B. Larson

**Affiliations:** 1 Division of Allergic Diseases, Mayo Clinic, 200 First Street SW, Rochester, MN 55905 USA; 2 Division of Epidemiology, Department of Quantitative Health Sciences, Mayo Clinic, Rochester, MN USA; 3 Division of Clinical Trials and Biostatistics, Department of Quantitative Health Sciences, Mayo Clinic, Rochester, MN USA; 4 Division of Allergy, Asthma and Clinical Immunology, Mayo Clinic, Scottsdale, AZ USA; 5 Department of Immunology, Mayo Clinic, Rochester, MN USA

**Keywords:** Chronic rhinosinusitis, Sinusitis, Mendelian randomization, Eosinophil, Neutrophil, Count, Risk, Genetics

## Abstract

**Background:**

Risk factors for the pathogenesis of chronic rhinosinusitis (CRS) remain largely undetermined, which is likely due to the heterogeneity of the disease. White blood cell counts have been largely unexplored as a risk factor for CRS even though different types of white blood cells are involved in the inflammatory process of CRS.

**Objective:**

To investigate causal associations between different types of white blood cells on risk of CRS utilizing a Mendelian randomization (MR) analysis.

**Methods:**

A two-sample MR analysis was performed using respective GWAS summary statistics for the exposure traits (neutrophil count, eosinophil count, basophil count, lymphocyte count, and monocyte count) and outcome trait (CRS). For the exposure traits, the European Bioinformatics Institute database of complete GWAS summary data was used. For the outcome trait, summary statistics for CRS GWAS were obtained from FinnGen. Primary analysis for MR was performed using inverse-variance weighted two-sample MR. Sensitivity analyses included weighted median, MR-Egger, and MR-PRESSO (raw and outlier-corrected).

**Results:**

Eosinophils were associated with CRS (OR = 1.55 [95% CI 1.38, 1.73]; p = 4.3E-14). Eosinophil results were similar across additional MR methods. MR results did not demonstrate significant causal relationships between neutrophils, lymphocytes, monocytes, or basophils with CRS. No significant pleiotropic bias was observed.

**Conclusions:**

In a two-sample MR analysis, a potential causal link between blood eosinophil counts and CRS has been demonstrated. In addition, causal relationships between blood counts among other white blood cell types and CRS were not found. Further studies involving genetic variation in CRS are needed to corroborate genetic causal effects for CRS.

**Supplementary Information:**

The online version contains supplementary material available at 10.1186/s13223-022-00739-2.

## Background

Chronic rhinosinusitis (CRS) is a heterogeneous disease, affecting 5–12% of the general population, characterized by chronic inflammation of the nose and paranasal sinuses. CRS is defined as the presence of two or more symptoms that include either nasal congestion/obstruction or nasal discharge, facial pain/pressure, and reduction/loss of smell that have been persistent for  ≥ 12 weeks along with objective evidence of nasal and sinus inflammation on nasal endoscopy or sinus computed tomography [1]. Given the direct costs of management of CRS in the United States of approximately $10 billion per year coupled with the indirect costs of loss of productivity and decreased quality of life, CRS represents an important public health issue [2].

Risk factors for the pathogenesis of CRS remain largely undetermined, which is likely due to the heterogeneity of the disease. Historically, CRS has been divided into two main clinical phenotypes based on the presence or absence of nasal polyps, with the majority of individuals (80%) with CRS not having nasal polyps [3, 4]. Other clinical CRS subgroups include aspirin exacerbated respiratory disease, allergic fungal sinusitis, and cystic fibrosis [5]. Since phenotypic categorization fails to account for the underlying inflammatory processes, [6] recent efforts have shifted to characterizing the heterogeneity of CRS into three different endotypes, Type 1, 2, and 3, that are based on specific immunologic mechanisms [7].

Potential risk factors for CRS may be broadly divided into demographics, environmental exposures, genetics, and premorbid or comorbid conditions [6]. Among all potential risk factors, only asthma and advancing age have shown to be risk factors consistently across multiple epidemiologic studies [1, 2, 6, 8]. Allergic rhinitis and smoking tobacco have also been implicated as risk factors in many studies, although some studies showed minimal increased risk [2, 6, 8]. Other studies investigating genetic variants, sex, race, air pollution, gastroesophageal reflux disease, and autoimmune diseases as risk factors have yielded mixed results [2, 6, 8]. The inconsistent results observed in these epidemiologic studies may be due in part to confounding and/or reverse causation.

White blood cell counts have been largely unexplored as a risk factor for CRS even though different types of white blood cells are involved in the inflammatory process of all three CRS endotypes. Given that white blood cell counts exhibit a fairly high level of heritability, we aimed to conduct a Mendelian randomization (MR) analysis to investigate the potential causal effects of white blood cell counts on risk of CRS. MR methodology is advantageous as it can limit the issues with confounding and reverse causation that occur in observational studies.

## Methods

This analysis was performed in accordance with the STROBE-MR reporting guidelines for MR studies (https://www.strobe-mr.org/) [9].

### Study design

We aimed to investigate causal associations between different types of white blood cells in relation to CRS utilizing MR. In brief, MR is an epidemiological technique that uses genetic variants to assess causal relationships in observational data [10, 11]. The utility of MR is based on Mendel’s laws whereby the natural random assignment of genetic variants during meiosis yields a random distribution of genetic variants in a population [12, 13]. Thereby, genetic variants that are related to an exposure of interest may be used as a surrogate of the exposure variable that is independent of unmeasured confounding, which is a considerable limitation of evidence from observational studies [11, 14]. Herein, we performed a two-sample MR analysis using respective GWAS summary statistics for the exposure traits (neutrophil count, eosinophil count, basophil count, lymphocyte count, and monocyte count) and outcome trait (CRS).

### MR assumptions

There are three core instrumental variable analysis assumptions relevant to MR studies: (1) Relevance—the SNP must be related to the exposure, (2) Independence—the SNP should not be correlated with confounders of the exposure-outcome relationship, and (3) Exclusion restriction—the genetic variant affects the outcome only through the exposure (i.e., no horizontal pleiotropy).

Relevance is ensured through strong association with the exposure of interest, since weak instruments can lead to biased estimates. To address this assumption, we restricted attention to genome-wide significant associations with the exposure, and calculated F-statistics for each SNP to quantify the strength of the instrument. Independence is not readily testable but can be justified by the random assortment of genetic variants at conception and accounting for potential confounding via population stratification via principal components adjustment in the respective exposure and outcome GWAS analyses. Exclusion restriction is addressed through various sensitivity analyses, including alternative MR methods robust to violations of this assumption as well as filtering out SNPs that may be related to other known risk factors.

### Data sources

For the exposure traits, we used the European Bioinformatics Institute (EBI) database of complete GWAS summary data that are publicly available from https://www.ebi.ac.uk/gwas/downloads/summary-statistics. The following study ID’s were used to obtain the exposure data: eosinophil count (ebi-a-GCST004606), neutrophil count (ebi-a-GCST004629), lymphocyte count (ebi-a-GCST004627), monocyte count (ebi-a-GCST004625), and basophil count (ebi-a-GCST004618). These GWAS analyses were conducted as described by Astle et al.[15] and are based on the UK Biobank, UK BiLEVE, and INTERVAL studies. Each of these GWAS datasets included  > 170,000 individuals and  > 29,000,000 SNPs.

Details regarding the UK Biobank, UK BiLEVE, and INTERVAL studies are described by Sudlow et al. [16], Wain et al. [17], and Moore et al.[18] respectively. In summary, UK Biobank is a population-based health research resource consisting of greater than 500,000 individuals, aged between 38 and 73 years, who were recruited between the years 2006 and 2010 from across the UK [19]. Participants provided a range of information via questionnaires and interviews that included demographics, health status, lifestyle measures, cognitive testing, personality self-report, and physical and mental health measures. Anthropometric measures, blood pressure readings and samples of blood, urine and saliva were also taken (data available at www.ukbiobank.ac.uk). A full description of the study design, participants and quality control methods have been described previously in detail [16, 20]. UK Biobank received ethical approval from the Research Ethics Committee (REC reference for UK Biobank is 11/NW/0382). The UK BiLEVE Study involved a subset of 50,008 participants of the UK Biobank study. The INTERVAL study is a prospective cohort study consisting of approximately 50,000 individuals, aged 18 years and older, who were recruited between the years 2012 and 2014 across England. Participants provided blood samples as well as information about height and weight, ethnicity, current smoking status, alcohol consumption, doctor-diagnosed anemia, medications, and menopausal status. A full description of the study design, protocol, and participants have been described previously [18]. The INTERVAL study was approved by the Cambridge (East) Research Ethics Committee. Informed consent was obtained from all participants of the UK Biobank, UK BiLEVE, and INTERVAL studies.

For the outcome trait, summary statistics for CRS GWAS were obtained from FinnGen Data Freeze 6 (study ID = finn-b-J10_CHRONSINUSITIS) that consists of  > 260,000 Finnish individuals and almost 17 M gene variants (data available at https://www.finngen.fi/en). FinnGen is a public—private partnership research project launched in Finland in 2017 that combines imputed genotype data generated from Finnish biobanks and Finnish digital health registries. Details regarding the study design, participants, genotyping, imputation and quality control methods have been described previously [21]. The definitions of FinnGen disease endpoints and their respective control definitions for each release are available at: https://www.finngen.fi/en/researchers/clinical-endpoints. FinnGen endpoints can also be obtained at https://risteys.finngen.fi/.

### Statistical analysis

Two-sample MR was performed using the R package TwoSampleMR, the corresponding R package to MR-Base [22]. The summary statistics for exposure and outcome traits were obtained using the included EBI database API. Extraction of instruments was performed under default settings for clumping and p-value thresholds (i.e., P-value  < 5e-08, clumping $${r}^{2}$$ cut-off = 0.001, clumping distance cut-off = 10,000 kb). Summary statistics were then harmonized to ensure agreement of effect alleles and remove ambiguous palindromic SNPs. To quantify the strength of the instruments, F-statistics were calculated by estimating the explained variation $${R}^{2}$$ per SNP using the equation $${R}^{2}=2*MAF*(1-MAF)*{\widehat{\beta }}^{2}$$ where MAF is the minor allele frequency and $$\widehat{\beta }$$ is the corresponding effect estimate. The final F-statistic was calculated via the following formula:

$$F=\frac{{R}^{2}\left(N-2\right)}{1-{R}^{2}}$$

where $N$ is the exposure GWAS sample size. F-statistics  < 10 were considered to be evidence of weak instruments.

Primary analysis for MR was performed using inverse-variance weighted (IVW) two-sample MR. Sensitivity analyses to account for potential violations of IV assumptions included weighted median, MR-Egger, and MR-PRESSO (raw and outlier-corrected). Additional quality assessments included funnel plots and assessment of pleiotropy by evaluating the MR-Egger regression intercept.

## Results

To assess the relationship of white blood cell counts (eosinophils, neutrophils, lymphocytes, monocytes, and basophils) with CRS a two-sample MR analysis was conducted. The number of SNPs that were eligible for each MR analysis for each endpoint was eosinophils (n = 160), neutrophils (n = 132), lymphocytes (n = 143), monocytes (n = 174), and basophils (n = 72). The distribution of the F-statistics for each IV is shown in Additional file 1: Fig. S1, indicating that evidence of weak instrument bias is low. The inverse variance weight MR results for each endpoint are shown in Table 1.

**Table 1:** Inverse variance weighted MR results

Exposure	OR	95% CI	P
Eosinophils	1.55	1.38–1.73	4.30E-14
Neutrophils	1.04	0.94–1.15	0.49
Lymphocytes	1.08	0.97–1.20	0.17
Monocytes	1.12	1.04–1.20	0.002
Basophils	1.12	0.97–1.29	0.12

*OR* odds ratio, *95% CI* 95% confidence interval

**Fig. 1:**
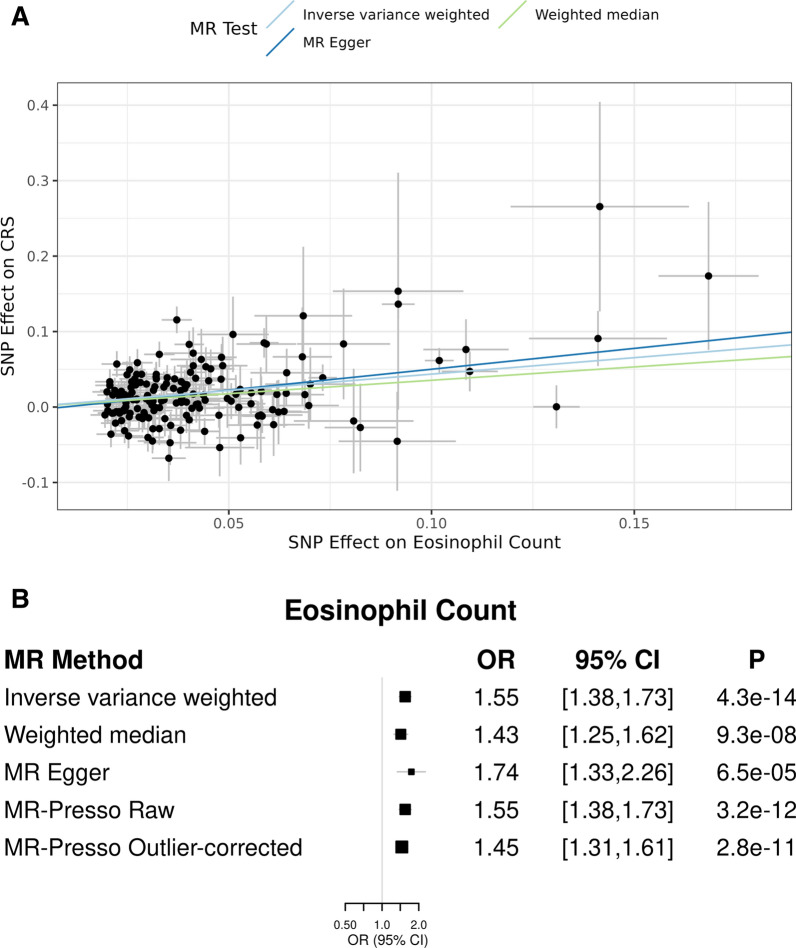
**A** SNP effect on eosinophil count and CRS. Two-sample MR analysis performed using GWAS summary statistics for eosinophil count (exposure trait) and CRS (outcome trait). Inverse variance weighted MR results: OR 1.55, 95% CI (1.38, 1.73), p = 4.3E-14. **B** SNP effect on eosinophil count and CRS. Two-sample MR analyses performed using GWAS summary statistics for eosinophil count (exposure trait) and CRS (outcome trait). Primary analysis was performed using inverse-variance weighted two-sample MR

Eosinophils were associated with CRS (OR = 1.55 [95% CI 1.38, 1.73]; p = 4.2E-14; Fig. 1A, B). Eosinophil results were similar across additional MR methods (Fig. 1B). MR results for neutrophils, lymphocytes, monocytes, and basophils are presented in Additional file 1: Figs. S2A–D and S3A–D. Monocyte counts were associated with CRS using the inverse variance weighted method (OR = 1.12 [95% CI 1.04, 1.20]; p = 0.002); however, these results were not consistent across MR methods (Additional file 1: Figs. S2C, S3C). The intercept from the Egger regression for each endpoint is presented in Additional file 2: Table S1. Funnel plots for each endpoint are shown Additional file 1: Fig. S4. No significant pleiotropic bias was observed.

## Discussion

Using MR analysis, this study provides evidence for a positive causal relationship between blood eosinophil counts and CRS. In addition, this study did not find evidence for causal relationships between blood counts among other white blood cell types and CRS. These findings are important, as they complement and build upon prior evidence that eosinophils play a key role in the pathophysiology of CRS even in the absence of nasal polyps. Furthermore, these results suggest that a potential causal role between neutrophils and CRS may need further investigation.

The MR approach has been widely used to determine potential causal relationships between various exposures (risk factors) and disease outcomes [15, 23, 24]. In regards to white blood cell counts, prior MR analyses have shown that eosinophil counts are causally related to several chronic diseases including asthma, celiac disease, rheumatoid arthritis, and type 1 diabetes [15]. Prior MR analyses also demonstrated that neutrophil counts were causally related to asthma, while lymphocyte counts had a protective effect on asthma and celiac disease and a causal effect on multiple sclerosis [15]. No causal effects were found between cardiometabolic outcomes (e.g., coronary heart disease, chronic kidney disease, type 2 diabetes) or neuropsychiatric diseases and any white blood cell counts except for lymphocyte counts having a causal effect on coronary heart disease [15]. A causal role for eosinophil counts and other white blood cell counts for CRS has not been previously reported.

From a mechanistic standpoint in terms of CRS, eosinophils are classically associated with type 2 inflammation that is characterized by elevated levels of IL-4, IL-5, IL-13, and total IgE. This type of eosinophil-associated inflammation has also been well-described in other allergic diseases [25, 26]. Despite differing CRS phenotypes distinguished by the presence or absence of nasal polyps, type 2 immune responses are clearly associated with disease severity regardless of phenotype. Individuals with CRS who exhibit a type 2 inflammatory response have increased symptom frequency, more severe clinical presentations, higher rates of long-term disease recurrence, and greater frequency of comorbid asthma [27]. These clinical observations accompany sinonasal biopsy findings that demonstrate evidence of eosinophilic inflammation with increased numbers of blood and tissue eosinophils, presence of eosinophil extracellular trap cell death, and deposition of Charcot-Leyden crystals [28].

As eosinophils are clearly involved with type 2 inflammation and increased disease severity, eosinophil counts have been studied as a potential biomarker to predict CRS clinical outcomes.^4^ Prior studies have demonstrated correlation between peripheral blood eosinophil counts with the number of eosinophils found in sinonasal tissue [29, 30]. However, the clinical utility of peripheral blood eosinophil counts as a biomarker is unclear. In one multicenter retrospective study involving  > 3000 subjects with CRS (both with and without nasal polyps), peripheral blood eosinophils > 10% were associated with greater risks of refractory and recurrent CRS [31]. This study only included subjects who had undergone surgery and excluded those with white blood cell counts  > 10,000/µl and those without pathologic specimens. Thus, selection bias and the retrospective nature are limitations of this study.

In terms of large clinical trials, the ability of blood eosinophil counts to predict treatment responses has also been indeterminate. In two large multicenter, randomized phase 3 trials using dupilumab to treat CRS and nasal polyps, dupilumab demonstrated significant benefit versus placebo regardless of the peripheral eosinophil count. Thereby, blood eosinophil counts provided no added specificity for identifying responsiveness to dupilumab [32]. In contrast, in a large multicenter, randomized phase 3 trial that used mepolizumab for treatment of CRS with nasal polyps, subgroup analyses suggested that efficacy of mepolizumab was greater in those with higher baseline blood eosinophil counts. However, low subject numbers in the lowest baseline blood eosinophil subgroup prevented a definitive conclusion to be made about the effect of eosinophil count on treatment response to mepolizumab [33, 34]. It is important to note that these clinical trials only included individuals with CRS with nasal polyps. A phase 3 clinical trial for those with CRS in the absence of nasal polyps is ongoing at the time of this manuscript. (dupilumab, ClinicalTrials.gov identifier: NCT04678856).

In contrast to the several clinical associations between eosinophil counts and CRS risk, studies involving genetic variants directly associated with eosinophil counts as a causal factor for CRS have been lacking. Prior studies looking at genetic risks for CRS have included several potential pathologic processes including gene variants involved in type 2 inflammation, chloride ion transport, human leukocyte antigens, innate immunity, tissue remodeling, and arachidonic acid metabolism [35]. With regards to Type 2 inflammation in CRS, for which eosinophils play a key role, previous studies have been limited to the genes that encode IL-4, IL-13, IL-33, and IL1RL1. One study by Yea et al. [36]. included 106 individuals with CRS (61 with nasal polyps) and found that the T/T allele at position -590 of the IL-4 gene conferred protection against CRS with nasal polyps. This finding has not been replicated. Palikhe et al.[37] studied 301 individuals with aspirin tolerant asthma and found no association between three IL-13 polymorphisms (SNPs rs1881457, rs1800925, rs20541) and CRS. In a study by Buysschaert et al.[38] that involved 273 individuals with CRS and nasal polyps and 415 controls, genetic variation in IL-33 (SNP rs3939286) was significantly associated with CRS with nasal polyps in a two-stage discovery and replication analysis (OR = 1.53 [95%CI 1.21,1.96]; p = 0.00041). In this same study, genetic variation in IL1RL1 (rs1420101), which is the receptor for IL-33, failed to associate with CRS and nasal polyps [38]. Two other studies of IL1RL1 genetic variation yielded mixed results [39, 40]. Overall, studies focused on genetic variation with genes related to type 2 inflammation have been few with little, if any, replication to confirm any findings. Furthermore, CRS phenotyping for inclusion into these studies has been variable and overrepresented with individuals with nasal polyps. Finally, none have investigated genetic variants associated with eosinophil counts themselves, as we performed in our study.

Although eosinophils have been closely linked to type 2 inflammation with CRS, neutrophils may also play an important role in the pathologic process. Neutrophilic infiltration and activation have been found to coexist with eosinophilic inflammation in those with severe type 2 CRS with nasal polyps. In these cases, increased neutrophil infiltration, independent of IL-17, significantly correlated with the presence of eosinophil extracellular trap cell death and deposition of Charcot-Leyden crystals [41]. Increased IL-8 production caused by Charcot-Leyden crystals appear to regulate neutrophil recruitment. In the CRS microenvironment, activated neutrophils secrete elastase and cathepsin G, both granule proteins with proteolytic activity linked to tissue remodeling, degradation of nasal epithelial barrier integrity, and increased mucus production [27]. Further studies are needed to better understand the role of neutrophils in CRS, especially in those with type 2 inflammation. Our MR analysis results indicate that neutrophils themselves do not have a direct causal role in CRS and suggest that neutrophil involvement may be secondary to other factors such as eosinophils, *S aureus* colonization, or other tissue-specific factors [27].

Our study has several strengths, including the use of large well-annotated data sources, two-sample MR analysis methodology, and multiple sensitivity analyses to account for genetic pleiotropy. Summary statistics were obtained from the UK Biobank and INTERVAL study (> 500 K individuals combined) which represent one the largest available sources of genetic associations with different types of white blood cell counts. Similarly, summary statistics were also obtained from FinnGen (> 260 K individuals) which is the largest available source of genetic associations with CRS. MR analyses are advantageous in their ability to limit potential issues of confounding and reverse causation that are often present in observational studies. In addition, the use of two-sample MR analysis allows for higher statistical power, especially with the large sample sizes used in this study. As horizontal pleiotropy can lead to confounding of MR estimates, consistency of the effect estimate derived from multiple sensitivity analyses (MR Egger regression, weighted median) provides greater assurance that the identified causal effect is valid.

Limitations of this study include the lack of available specific lymphocyte subset summary statistics, CRS diagnosis methodology in FinnGen, and generalizability of the findings. When assessing the potential role of lymphocyte counts in CRS, the summary statistics used in our study were based on a total lymphocyte population. As specific B cell and T cell lymphocyte populations have been shown to play important roles in the pathophysiology of several allergic conditions including asthma [42, 43], our study was not able to address the potential role of selected lymphocyte populations in CRS. If summary statistics were available for lymphocyte subsets, our study may have yielded different results in terms of a lymphocyte role in CRS. In regards to FinnGen methodology, the clinical diagnostic endpoints in FinnGen are derived from an algorithm based on ICD codes that include or exclude specific diagnoses. Utilizing data from electronic health records can be challenging. Diagnostic codes may not be used consistently, and the meaning of any given diagnostic code may vary among different providers and over time [44]. Even so, prior work has demonstrated that diagnostic codes can be employed effectively to accurately identify CRS for research purposes [45]. In terms of generalizability, genetic associations were estimated from primarily individuals of European descent, and thus our results may not be applicable to individuals of other ethnicities. Genetic variants associated with white blood cell counts have been explored in different ethnic populations [46], but available summary data regarding genetic variants associated with CRS is almost nonexistent other than FinnGen. Additional studies are needed to investigate causal genetic factors of CRS.

## Conclusions

In a two-sample MR analysis, we demonstrate evidence for a potential causal link between blood eosinophil counts and CRS. In addition, causal relationships between blood counts among other white blood cell types and CRS were not found. These findings support observations of eosinophil involvement in type 2 inflammation in the pathophysiology of CRS. However, a potential causal role between neutrophils and CRS remains unclear. Further studies involving genetic variation in CRS are needed to corroborate the genetic causal effects found in our investigation.

## Supplementary Information

**Additional file 1: Figure S1.** F-statistics of IV SNPs used in MR Analysis. **Figure S2.** A SNP effect on neutrophil count and CRS. Two-sample MR analyses performed using GWAS summary statistics for neutrophil count (exposure trait) and CRS (outcome trait). Primary analysis was performed using inverse-variance weighted two-sample MR. B SNP effect on lymphocyte count and CRS. Two-sample MR analyses performed using GWAS summary statistics for lymphocyte count (exposure trait) and CRS (outcome trait). Primary analysis was performed using inverse-variance weighted two-sample MR. C SNP effect on monocyte count and CRS. Two-sample MR analyses performed using GWAS summary statistics for monocyte count (exposure trait) and CRS (outcome trait). Primary analysis was performed using inverse-variance weighted two-sample MR. D SNP effect on basophil count and CRS. Two-sample MR analyses performed using GWAS summary statistics for basophil count (exposure trait) and CRS (outcome trait). Primary analysis was performed using inverse-variance weighted two-sample MR. **Figure S3.** A SNP effect on neutrophil count and CRS. Two-sample MR analysis performed using GWAS summary statistics for neutrophil count (exposure trait) and CRS (outcome trait). Inverse variance weighted MR results: OR 1.04, 95% CI (0.94, 1.15), p=0.49. B SNP effect on lymphocyte count and CRS. Two-sample MR analysis performed using GWAS summary statistics for lymphocyte count (exposure trait) and CRS (outcome trait). Inverse variance weighted MR results: OR 1.08, 95% CI (0.97, 1.20), p=0.17. C SNP effect on monocyte count and CRS. Two-sample MR analysis performed using GWAS summary statistics for monocyte count (exposure trait) and CRS (outcome trait). Inverse variance weighted MR results: OR 1.12, 95% CI (1.04, 1.20), p=0.002. D SNP effect on basophil count and CRS. Two-sample MR analysis performed using GWAS summary statistics for basophil count (exposure trait) and CRS (outcome trait). Inverse variance weighted MR results: OR 1.12, 95% CI (0.97, 1.29), p=0.12.**Figure S4.** Funnel plots for white blood cell types.

**Additional file 2: Table S1.** Egger intercept test.

## Data Availability

The datasets analysed during the current study are available in the European Bioinformatics Institute database, [https://www.ebi.ac.uk/gwas/downloads/summary-statistics], and in the FinnGen repository [https://www.finngen.fi/en].

## Abbreviations

CRS: Chronic rhinosinusitis
EBI: European Bioinformatics Institute
GWAS: Genome-wide association studies
IL: Interleukin
IV: Inverse variance
MAF: Minor allele frequency
MR: Mendelian randomization
SNP: Single nucleotide polymorphism
