# Supplementary material for: White blood cells and chronic rhinosinusitis: a Mendelian randomization study
Source: Allergy Asthma Clin Immunol. 2022 Nov 22;18:98. doi: 10.1186/s13223-022-00739-2 (PMC9682667; doi:10.1186/s13223-022-00739-2)
Supplement: Supplementary file 1 — Additional file 1: Figure S1. F-statistics of IV SNPs used in MR Analysis. Figure S2. A SNP effect on neutrophil count and CRS. Two-sample MR analyses performed using GWAS summary statistics for neutrophil count (exposure trait) and CRS (outcome trait). Primary analysis was performed using inverse-variance weighted two-sample MR. B SNP effect on lymphocyte count and CRS. Two-sample MR analyses performed using GWAS summary statistics for lymphocyte count (exposure trait) and CRS (outcome trait). Primary analysis was performed using inverse-variance weighted two-sample MR. C SNP effect on monocyte count and CRS. Two-sample MR analyses performed using GWAS summary statistics for monocyte count (exposure trait) and CRS (outcome trait). Primary analysis was performed using inverse-variance weighted two-sample MR. D SNP effect on basophil count and CRS. Two-sample MR analyses performed using GWAS summary statistics for basophil count (exposure trait) and CRS (outcome trait). Primary analysis was performed using inverse-variance weighted two-sample MR. Figure S3. A SNP effect on neutrophil count and CRS. Two-sample MR analysis performed using GWAS summary statistics for neutrophil count (exposure trait) and CRS (outcome trait). Inverse variance weighted MR results: OR 1.04, 95% CI (0.94, 1.15), p=0.49. B SNP effect on lymphocyte count and CRS. Two-sample MR analysis performed using GWAS summary statistics for lymphocyte count (exposure trait) and CRS (outcome trait). Inverse variance weighted MR results: OR 1.08, 95% CI (0.97, 1.20), p=0.17. C SNP effect on monocyte count and CRS. Two-sample MR analysis performed using GWAS summary statistics for monocyte count (exposure trait) and CRS (outcome trait). Inverse variance weighted MR results: OR 1.12, 95% CI (1.04, 1.20), p=0.002. D SNP effect on basophil count and CRS. Two-sample MR analysis performed using GWAS summary statistics for basophil count (exposure trait) and CRS (outcome trait). Inverse variance weighted MR resul [file 13223_2022_739_MOESM1_ESM.pptx]

## Slide 1
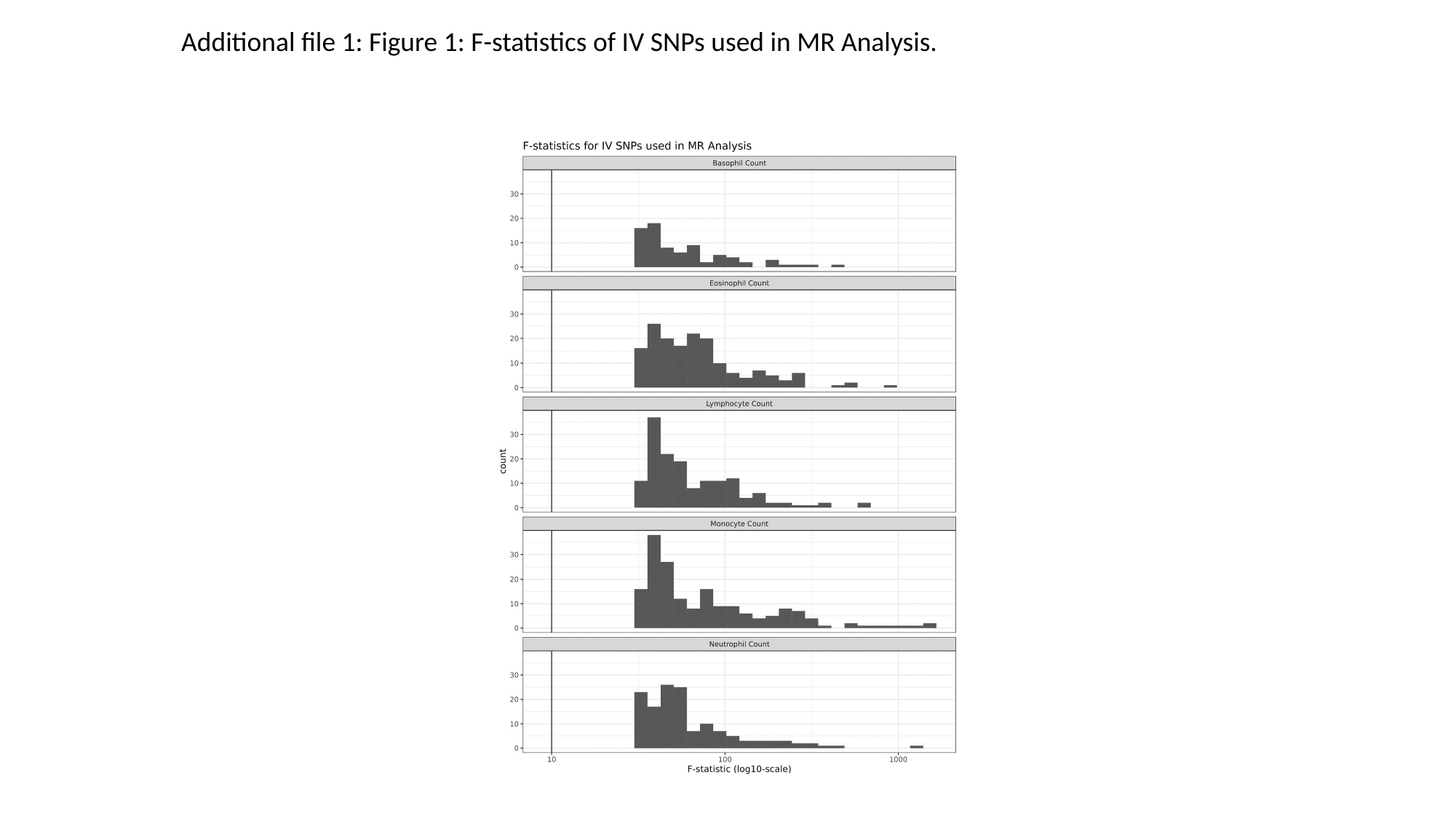

Additional file 1: Figure 1: F-statistics of IV SNPs used in MR Analysis.

## Slide 2
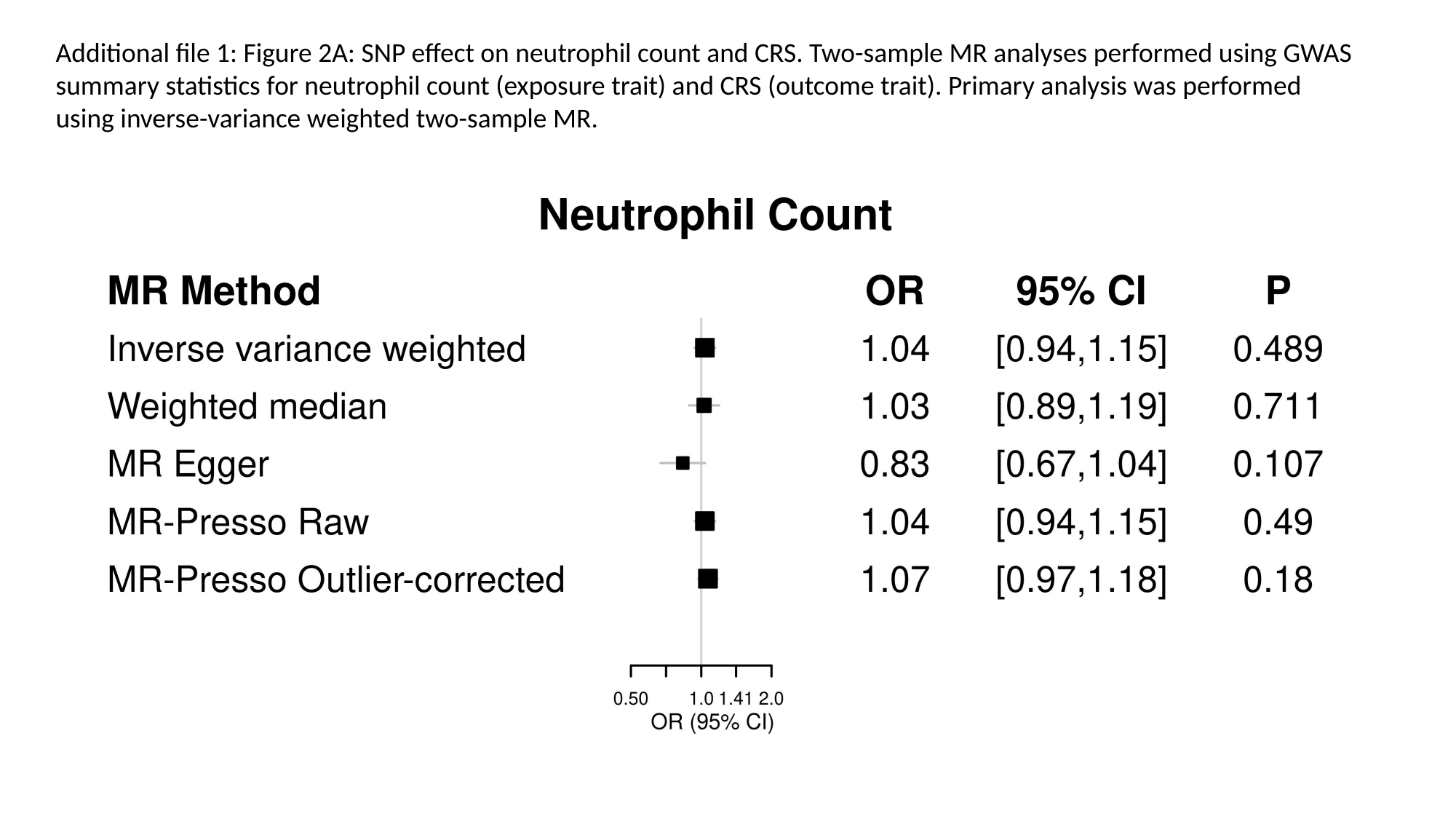

Additional file 1: Figure 2A: SNP effect on neutrophil count and CRS. Two-sample MR analyses performed using GWAS summary statistics for neutrophil count (exposure trait) and CRS (outcome trait). Primary analysis was performed using inverse-variance weighted two-sample MR.

## Slide 3
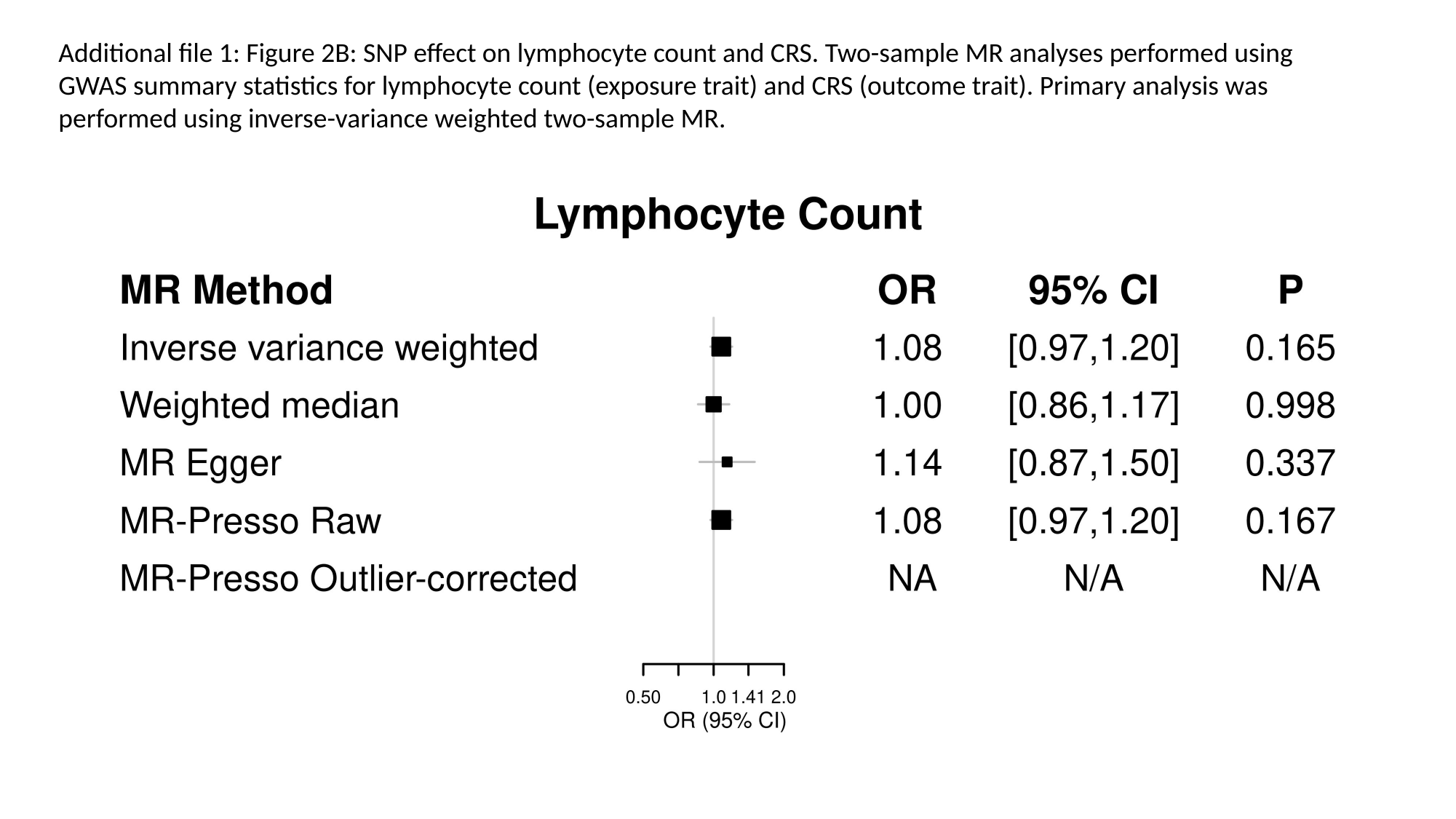

Additional file 1: Figure 2B: SNP effect on lymphocyte count and CRS. Two-sample MR analyses performed using GWAS summary statistics for lymphocyte count (exposure trait) and CRS (outcome trait). Primary analysis was performed using inverse-variance weighted two-sample MR.

## Slide 4
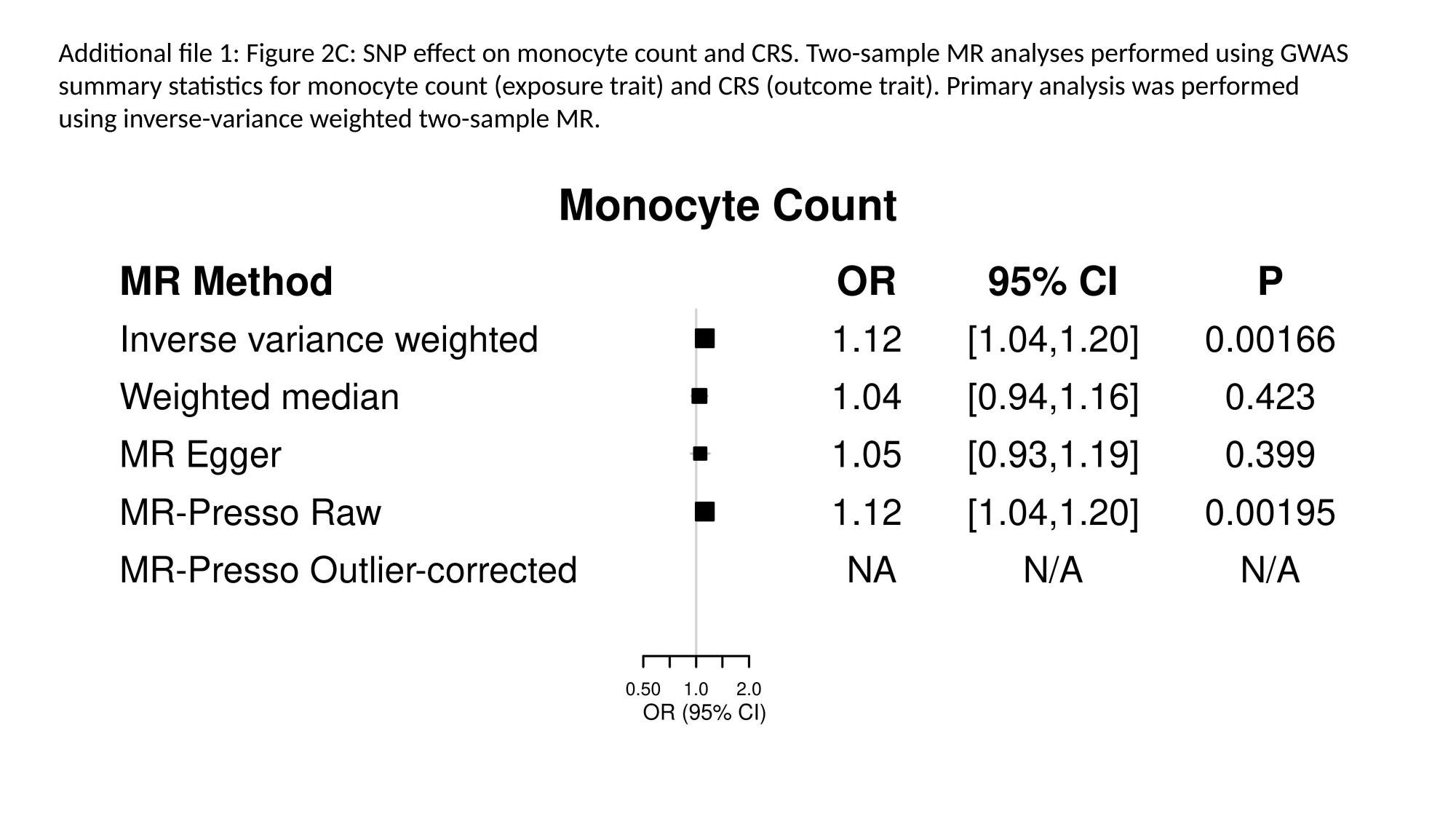

Additional file 1: Figure 2C: SNP effect on monocyte count and CRS. Two-sample MR analyses performed using GWAS summary statistics for monocyte count (exposure trait) and CRS (outcome trait). Primary analysis was performed using inverse-variance weighted two-sample MR.

## Slide 5
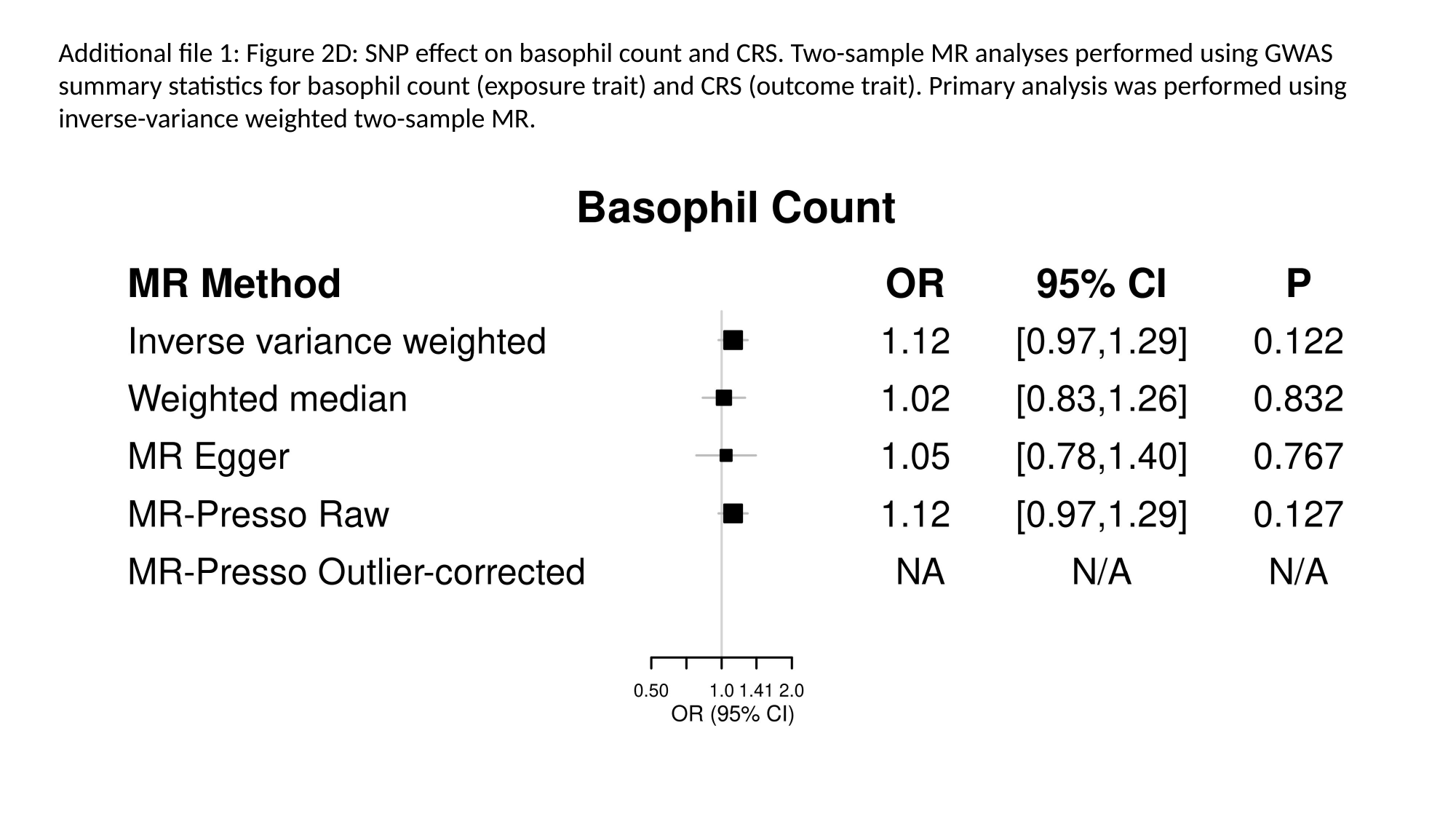

Additional file 1: Figure 2D: SNP effect on basophil count and CRS. Two-sample MR analyses performed using GWAS summary statistics for basophil count (exposure trait) and CRS (outcome trait). Primary analysis was performed using inverse-variance weighted two-sample MR.

## Slide 6
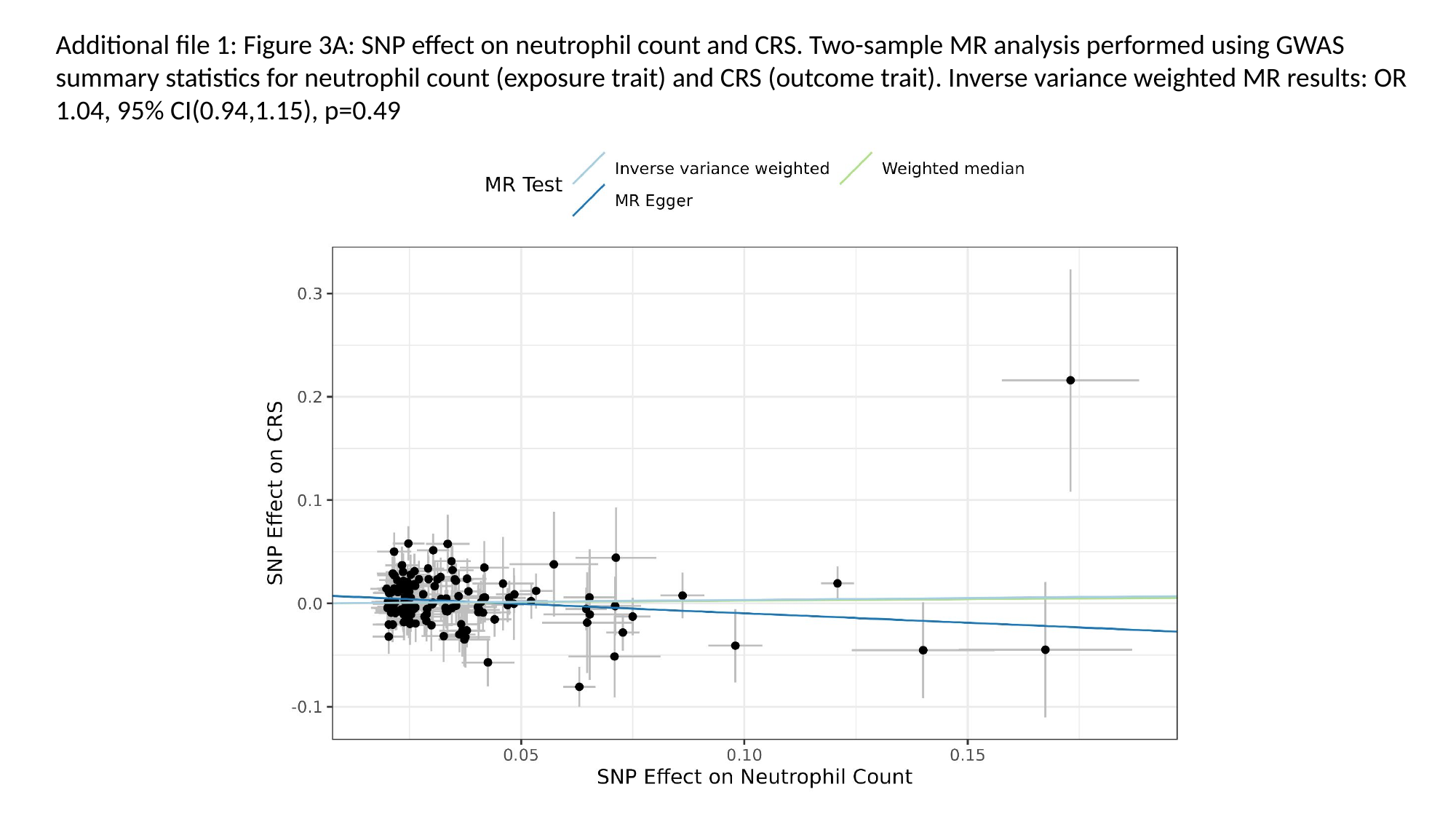

Additional file 1: Figure 3A: SNP effect on neutrophil count and CRS. Two-sample MR analysis performed using GWAS summary statistics for neutrophil count (exposure trait) and CRS (outcome trait). Inverse variance weighted MR results: OR 1.04, 95% CI(0.94,1.15), p=0.49

## Slide 7
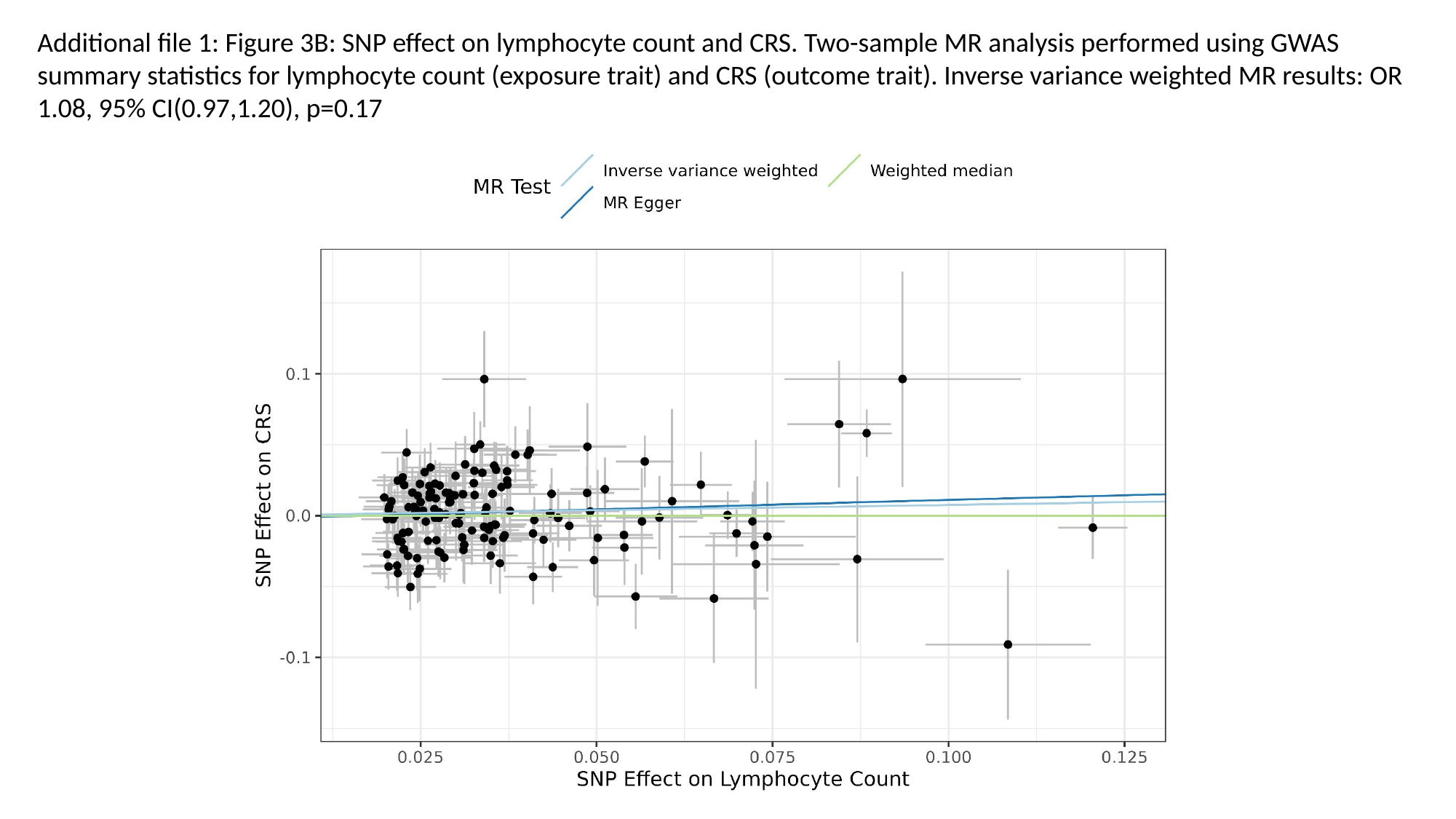

Additional file 1: Figure 3B: SNP effect on lymphocyte count and CRS. Two-sample MR analysis performed using GWAS summary statistics for lymphocyte count (exposure trait) and CRS (outcome trait). Inverse variance weighted MR results: OR 1.08, 95% CI(0.97,1.20), p=0.17

## Slide 8
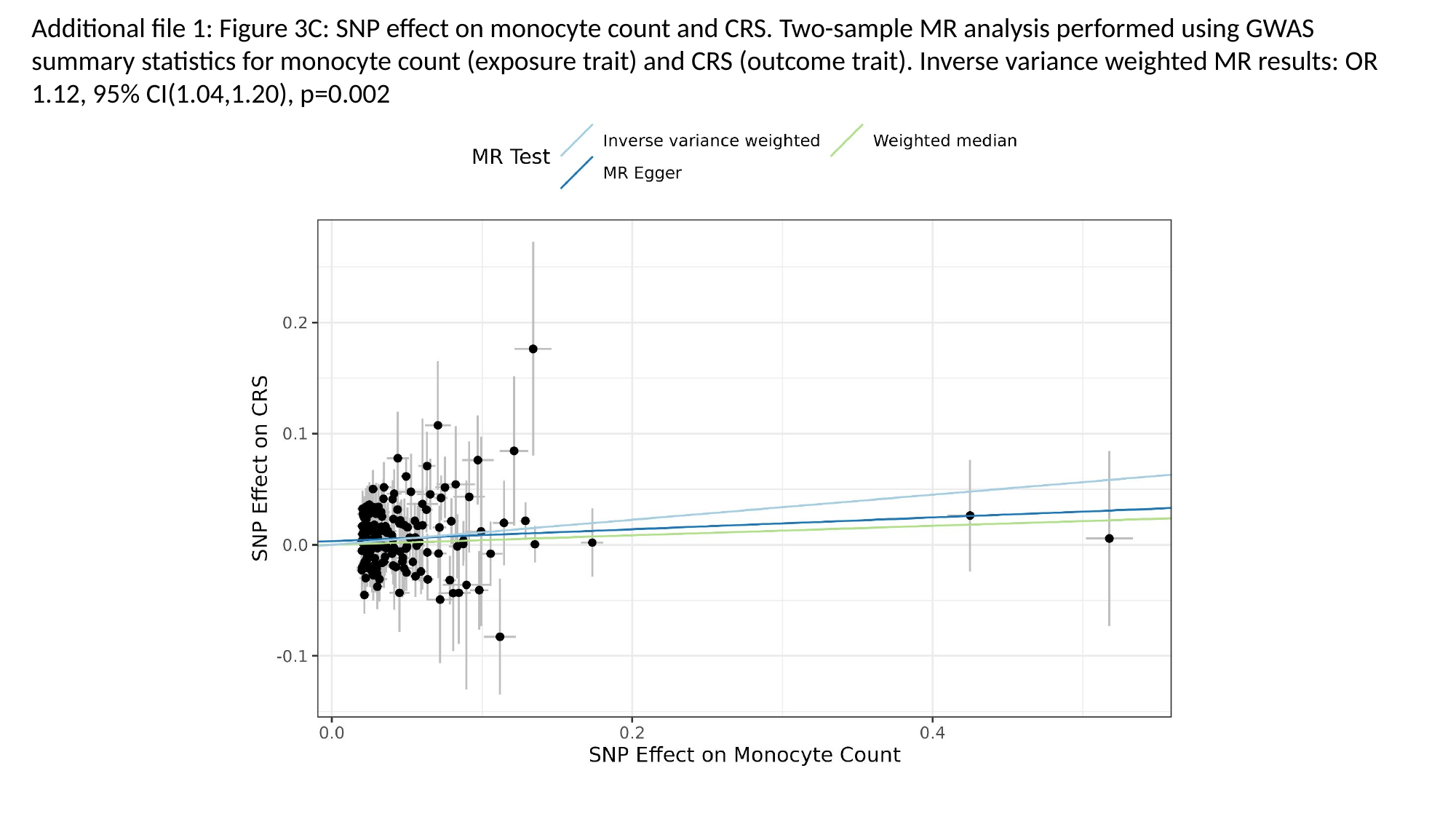

Additional file 1: Figure 3C: SNP effect on monocyte count and CRS. Two-sample MR analysis performed using GWAS summary statistics for monocyte count (exposure trait) and CRS (outcome trait). Inverse variance weighted MR results: OR 1.12, 95% CI(1.04,1.20), p=0.002

## Slide 9
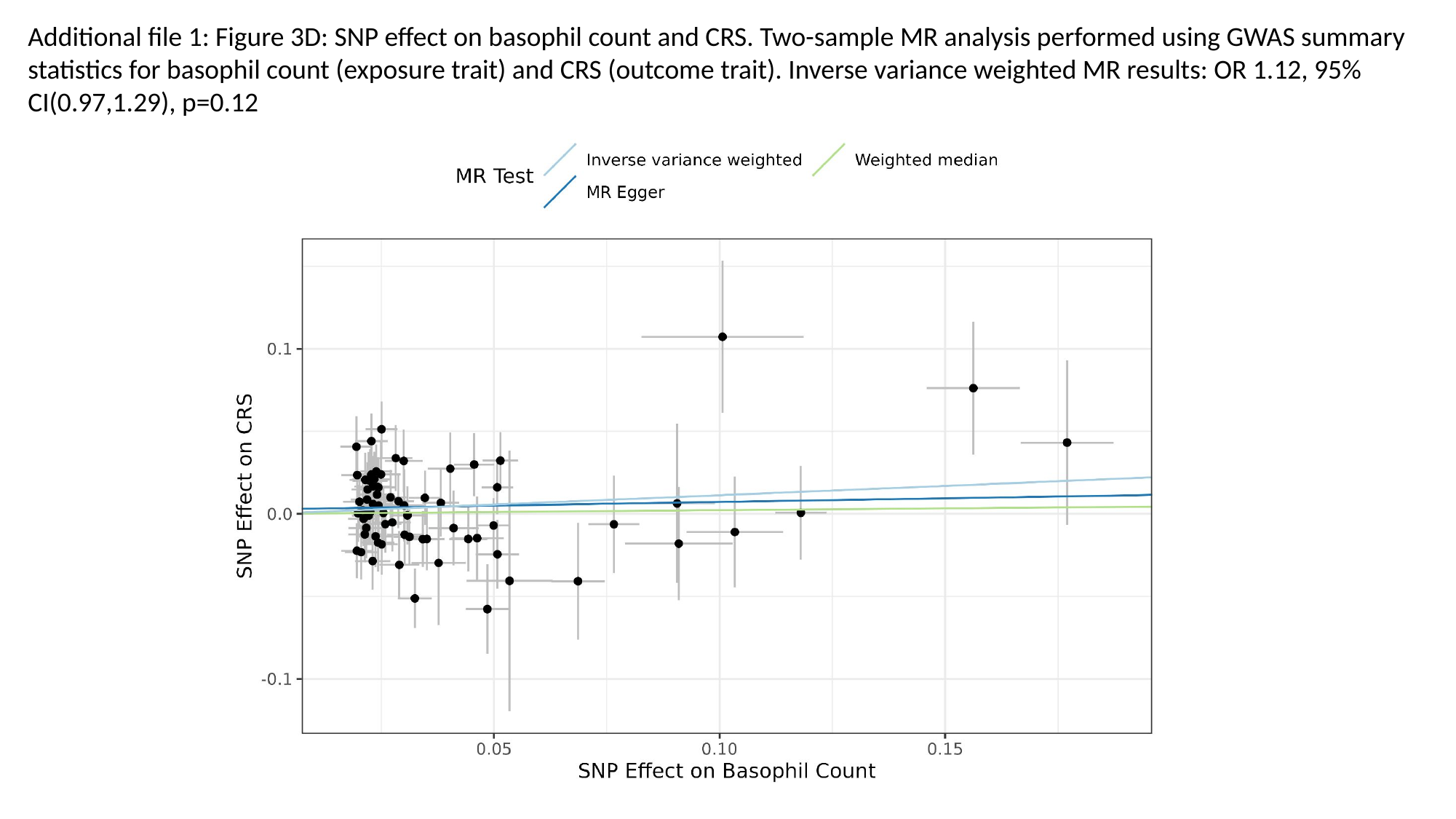

Additional file 1: Figure 3D: SNP effect on basophil count and CRS. Two-sample MR analysis performed using GWAS summary statistics for basophil count (exposure trait) and CRS (outcome trait). Inverse variance weighted MR results: OR 1.12, 95% CI(0.97,1.29), p=0.12

## Slide 10
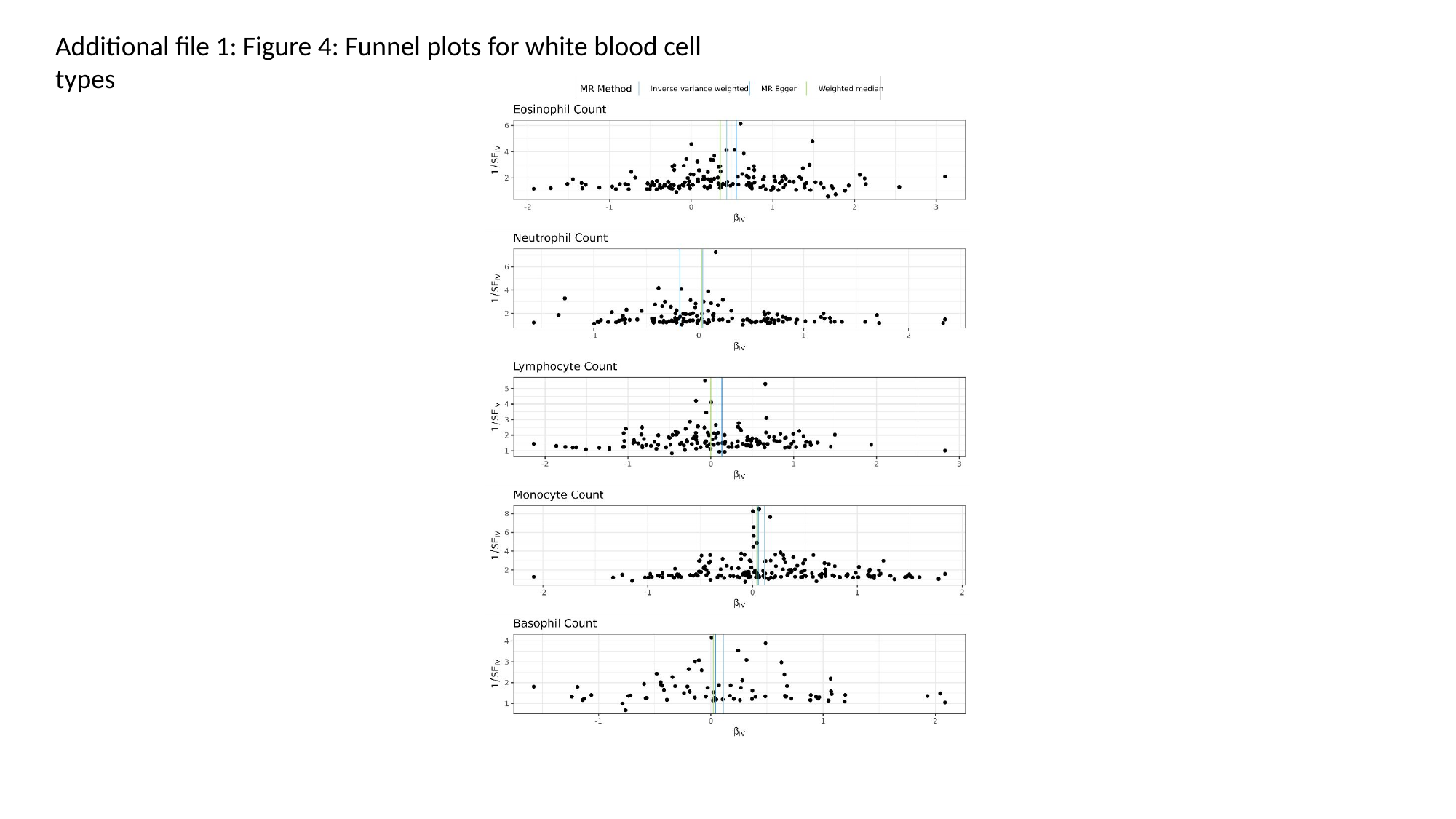

Additional file 1: Figure 4: Funnel plots for white blood cell types
