# Supplementary material for: White blood cells and chronic rhinosinusitis: a Mendelian randomization study
Source: Allergy Asthma Clin Immunol. 2022 Nov 22;18:98. doi: 10.1186/s13223-022-00739-2 (PMC9682667; doi:10.1186/s13223-022-00739-2)
Supplement: Supplementary file 2 — Additional file 2: Table S1. Egger intercept test. [file 13223_2022_739_MOESM2_ESM.docx]

Additional file 2: Table 1: Egger intercept test

| Exposure | Egger intercept | SE | P |
| --- | --- | --- | --- |
| eosinophils | -0.0052 | 0.0055 | 0.34 |
| neutrophils | 0.0087 | 0.004 | 0.031 |
| lymphocytes | -0.0023 | 0.005 | 0.65 |
| monocytes | 0.0034 | 0.0029 | 0.25 |
| basophils | 0.0028 | 0.0053 | 0.6 |

SE = standard error
